# High affinity binding of H3K14ac through collaboration of bromodomains 2, 4 and 5 is critical for the molecular and tumor suppressor functions of PBRM1

**DOI:** 10.1002/1878-0261.12434

**Published:** 2019-02-02

**Authors:** Lili Liao, Nilda L. Alicea‐Velázquez, Lauren Langbein, Xiaohua Niu, Weijia Cai, Eun‐Ah Cho, Meiling Zhang, Celeste B. Greer, Qin Yan, Michael S. Cosgrove, Haifeng Yang

**Affiliations:** ^1^ Department of Pathology, Anatomy and Cell Biology Thomas Jefferson University Philadelphia PA USA; ^2^ Department of Pathology Yale University New Haven CT USA; ^3^ Department of Biochemistry and Molecular Biology State University of New York Upstate Medical University Syracuse NY USA; ^4^ Department of Chemistry and Biochemistry Central Connecticut State University New Britain CT USA; ^5^ Department of Gastrointestinal Surgery The Sixth Affiliated Hospital of Guangzhou Medical University China; ^6^ Fox Chase Cancer Center Philadelphia PA USA; ^7^ Department of Pharmacology Vanderbilt University Nashville TN USA

**Keywords:** bromodomain, H3K14ac, kidney cancer, PBRM1, synergy

## Abstract

Polybromo‐1 (PBRM1) is an important tumor suppressor in kidney cancer. It contains six tandem bromodomains (BDs), which are specialized structures that recognize acetyl‐lysine residues. While BD2 has been found to bind acetylated histone H3 lysine 14 (H3K14ac), it is not known whether other BDs collaborate with BD2 to generate strong binding to H3K14ac, and the importance of H3K14ac recognition for the molecular and tumor suppressor function of PBRM1 is also unknown. We discovered that full‐length PBRM1, but not its individual BDs, strongly binds H3K14ac. BDs 2, 4, and 5 were found to collaborate to facilitate strong binding to H3K14ac. Quantitative measurement of the interactions between purified BD proteins and H3K14ac or nonacetylated peptides confirmed the tight and specific association of the former. Interestingly, while the structural integrity of BD4 was found to be required for H3K14ac recognition, the conserved acetyl‐lysine binding site of BD4 was not. Furthermore, simultaneous point mutations in BDs 2, 4, and 5 prevented recognition of H3K14ac, altered promoter binding and gene expression, and caused PBRM1 to relocalize to the cytoplasm. In contrast, tumor‐derived point mutations in BD2 alone lowered PBRM1's affinity to H3K14ac and also disrupted promoter binding and gene expression without altering cellular localization. Finally, overexpression of PBRM1 variants containing point mutations in BDs 2, 4, and 5 or BD2 alone failed to suppress tumor growth in a xenograft model. Taken together, our study demonstrates that BDs 2, 4, and 5 of PBRM1 collaborate to generate high affinity to H3K14ac and tether PBRM1 to chromatin. Mutations in BD2 alone weaken these interactions, and this is sufficient to abolish its molecular and tumor suppressor functions.

**Abbreviations**
3m: N263A‐DLF>AAA‐N739A mutations
BD2*: N263A mutation
BD2IFD: in‐frame deletion of N263
BD4*: DLF>AAA mutation
BD5*: N739A mutation
BD: bromodomain
BLI: biolayer interferometry
ccRCC: clear cell renal cell carcinoma
CTLA‐4: cytotoxic T lymphocyte‐associated 4
H3K14ac: acetylated histone H3 lysine 14
HIF: hypoxia‐inducible factor
mRCC: metastatic renal cell carcinoma
PBAF: polybromo‐1‐associated BRG1 or BRM‐associated factor
PBRM1: polybromo‐1
PD‐1: programmed death 1
PD‐L1: programmed death ligand 1

## 1 Introduction

Approximately 75% of the renal cell carcinomas are of the clear cell type (ccRCC). Among them, 70–80% of sporadic ccRCC tumors harbor biallelic inactivation of *VHL* (Linehan *et al*., 2004). pVHL, the protein product of the *VHL* tumor suppressor gene, is a component of an E3 ubiquitin ligase complex. This complex promotes the poly‐ubiquitylation and proteasomal degradation of the α subunits of the heterodimeric transcription factor hypoxia‐inducible factor (HIF). When pVHL is inactivated by various mechanisms in ccRCC, HIF becomes constitutively activated and turns on the hypoxia response transcriptome. This subsequently drives tumorigenesis and growth of ccRCC tumors (Kaelin, 2005).

Most solid tumors harbor multiple driver mutations in cancer genes to achieve the hallmarks of cancer (Hanahan and Weinberg, 2011; Vogelstein *et al*., 2013). Forty‐one percent of ccRCC tumors have inactivating mutations in the *PBRM1* gene, and they occur throughout the coding sequence (Varela *et al*., 2011). The encoded polybromo‐1 (PBRM1) protein is a specificity subunit of the PBRM1‐associated BRG1 or BRM‐associated factor (PBAF) chromatin‐remodeling complex (Varela *et al*., 2011). The PBAF complex utilizes the energy of ATP hydrolysis to regulate DNA accessibility by inserting, removing, or sliding nucleosomes (Hargreaves and Crabtree, 2011). The high mutation rate of *PBRM1* in ccRCC has been confirmed by multiple studies (Cancer Genome Atlas Research Network, 2013; Dalgliesh *et al*., 2010; Guo *et al*., 2012; Pena‐Llopis *et al*., 2012; Sato *et al*., 2013). Mutations in other genes such as *BAP1*,* SETD2*,* KDM5C*/*JARID1C*,* PTEN,* and *UTX* have also been identified, but their mutation rates are much lower than that of *PBRM1* (Liao *et al*., 2015).

*PBRM1* is a key tumor suppressor in ccRCC. Its mutations are predominantly loss‐of‐function mutations. Suppression of PBRM1 leads to changes in pathways regulating chromosome instability and cell proliferation (Varela *et al*., 2011). Genomic sequencing of multiple foci of ccRCC tumors has revealed that the majority of *PBRM1* mutations occur early in tumorigenesis, while mutations in the other secondary tumor suppressor genes occur later during tumor development (Gerlinger *et al*., 2012, 2014). A *PBRM1* germline mutation was recently reported to predispose patients to ccRCC (Benusiglio *et al*., 2015). PBRM1‐deficient cancer cells exhibit a weakened HIF transcriptional signature (Gao *et al*., 2017). In mice, kidney‐specific deletion of either *Vhl* or *Pbrm1* does not lead to ccRCC, but their combined loss does (Gu *et al*., 2017; Nargund *et al*., 2017). Thus, PBRM1 restrains HIF's tumor‐promoting activity.

Since 2005, vascular endothelial growth factor receptor tyrosine kinase inhibitors have been the cornerstone of therapy for metastatic renal cell carcinoma (mRCC) (Choueiri and Motzer, 2017; Mihaly *et al*., 2012). Recently, however, immunotherapy checkpoint inhibitors that target programmed death 1 (PD‐1), programmed death ligand 1 (PD‐L1), and cytotoxic T lymphocyte‐associated 4 (CTLA‐4) have gained traction in mRCC. Indeed, RCC experts are declaring this combination the new standard of care in the treatment of mRCC (Chustecka, 2017; Rini, 2017). Loss‐of‐function mutations of PBRM1 were recently discovered to be associated with response to checkpoint inhibition therapies in ccRCC patients treated with anti‐PD‐1 alone or in combination with anti‐CTLA‐4 therapies (Miao *et al*., 2018). In addition, PBRM1 mutations were found to enhance T‐cell‐mediated killing of tumor cells in an unbiased, high‐throughput screen in a melanoma model (Pan *et al*., 2018). Thus, mutation of PBRM1 is a biomarker for immunotherapy response.

PBRM1 contains six tandem bromodomains (BDs), BD1–6, from the N terminus to the middle of the protein. BDs are small domains with specific affinity for *N*‐ε‐lysine acetylation on histones and other proteins (Dhalluin *et al*., 1999; Owen *et al*., 2000). However, it is not clear why PBRM1 contains so many BDs while most other BD‐containing proteins have only one or two BDs. As PBRM1 is a specificity subunit of PBAF, it is hypothesized that it binds to lysine‐acetylated histone tails. Previous analyses revealed that BD2 preferentially recognizes acetylated lysine 14 of histone H3 (H3K14ac) and potentially other acetylation sites on histone H3 (Chandrasekaran and Thompson, 2007; Charlop‐Powers *et al*., 2010; Thompson, 2009). Recently, Porter and Dykhuizen (2017) confirmed that while BD2 is critical for H3K14ac binding, mutation of BD2 only moderately reduced PBRM1's binding to H3K14ac and its association with chromatin. These findings prompted the authors to speculate that the other BDs bind additional acetylated histone sites to ensure tight chromatin association (Porter and Dykhuizen, 2017). Slaughter *et al*. (2018) confirmed the binding to H3K14ac by BD2 BD4 and showed that neighboring BDs variably influence nucleosome interactions and cellular function. However, it is still not clear how critical H3K14ac recognition is to PBRM1's association with the chromatin and how mutant PBRM1 impacts tumor growth in a mouse model.

In this study, we discovered that BDs 2, 4, and 5 collaborate to generate high affinity binding to H3K14ac. Simultaneous mutations in the involved BDs were found to completely abolish H3K14ac recognition, causing PBRM1 to detach from chromatin and relocalize to the cytoplasm. In addition, tumor‐derived mutations in BD2 resulted in reduced recognition of H3K14ac by PBRM1. Although these mutations failed to significantly change PBRM1's binding to chromatin, they significantly impaired PBRM1's ability to bind to promoters and regulate gene expression and severely crippled PBRM1's tumor suppressor function. Altogether, our findings suggest that BDs 2, 4, and 5 play a central role in PBRM1 molecular and biological functions by mediating high‐affinity recognition of H3K14ac.

## 2 Materials and methods

### 2.1 Cell culture

HEK293T, HeLa, and 786‐O cells were purchased from American Type Culture Collection and cultured in Dulbecco's modified Eagle's medium supplemented with 10% FBS. The cell lines were maintained in incubators at 37 °C with 5% CO_2_.

### 2.2 Protein expression and purification

Polybromo‐1 constructs consisting of BD2, BD4, BD234, and BD245 were subcloned into pGST parallel expression vectors (Sheffield *et al*., 1999) individually expressed in *Escherichia coli* strain BL21, purified over a GST column (GE Healthcare, Pittsburgh, PA, USA; Catalog #17‐5130‐01), eluted with 10 mm reduced glutathione, and dialyzed against 2 L of dialysis buffer (25 mm Tris/HCl, 500 mm NaCl, pH 8.0) to remove glutathione. The purified proteins were incubated with 100 units of rTEV (Invitrogen, Carlsbad, CA, USA; Catalog #10127‐017) for 36 hr at 4 °C. The GST protein and rTEV were removed by passing the purified proteins over a GST column, and the flow‐through was collected for biolayer interferometry (BLI).

### 2.3 Biolayer interferometry (BLI)

Histone H3 binding was measured using BLI using a ForteBio Octet Red instrument (San Jose, CA, USA). The assay buffer consisted of 20 mm Tris (pH 7.5), 300 mm NaCl, 1 mm tris(2‐carboxyethyl)phosphine, 1 μm ZnCl_2_, 10% glycerol, and 0.5 mg·mL^−1^ bovine serum albumin. BD2, BD4, BD234, and BD245 proteins were prepared at a concentration of 1 or 2 μm, and the biotin‐labeled H3 peptide was prepared at a concentration of 25 nm. Biotinylated H3 peptide was immobilized onto streptavidin‐coated sensors (ForteBio) until a threshold signal of 0.4 nm was reached. BD2, BD4, BD234, and BD245 were allowed to associate with the ligand‐bound sensor for 2000 s followed by dissociation with assay buffer for 2000 s. All steps were performed at 25 °C with sensors dipped into 200 μL of sample and stirred at 100 ***g***. Reference sensors (without H3 peptide) were used for each variant tested to correct for background binding and baseline drift. Data were processed using the fortebio data analysis program (version 6.4) utilizing reference subtraction and Savitzky‐Golay filtering. Data were plotted using prismgraph (Graphpad, San Diego, CA, USA).

### 2.4 Plasmids

Full‐length PBRM1 cDNA was generated by reverse transcription–PCR from 786‐O cells and cloned into 3XFlag‐CMV10 (Sigma, St. Louis, MO, USA) and pLNCX‐GFP vectors (gift from X. Wei (Wang *et al*., 2014)). Internal deletion, truncation, and point mutations were made with PCR and the QuikChange Site‐Direct Mutagenesis kit (Agilent Technologies, Santa Clara, CA, USA; Catalog #200523) and verified by DNA sequencing. PBRM1 lentiviral shRNA vectors were purchased from Sigma. The sequences were PBRM1‐sh94: CCGGAGTCTTTGATCTACAAA; PBRM1‐sh890: CCGGAATGCCAGGCACTATA.

### 2.5 Peptide pull‐down, immunoblotting, mono‐nucleosome pull‐down

Biotinylated histone H3 peptides were synthesized by Anaspec (Fremont, CA, USA) and contained residues 1–21 of histone H3 (ARTKQTARKSTGGKAPRKQLT). Overexpressed Flag‐PBRM1 in HEK293T cells or endogenous PBRM1 from 786‐O cells were extracted with high salt buffer (containing 0.42 m NaCl). The samples were mixed with unmodified or modified biotinylated histone H3 peptides and incubated at 4 °C for 1 hr, followed by addition of streptavidin beads (Pierce Biotechnology, Waltham, MA, USA; Catalog #20347) and incubation at 4 °C for 1 hr. The bound materials were washed three times with NETN buffer (100 mm NaCl, 20 mm Tris/Cl pH 8.0, 0.5 mm EDTA, 0.5% (v/v) Nonidet P‐40) and examined by western blots. Cell lysates or peptide pull‐down products were loaded and resolved by SDS/PAGE. After electrophoresis, proteins were transferred to nitrocellulose membranes. The membranes were blocked at room temperature for 1 hr, then incubated with appropriate primary antibodies overnight at 4 °C, and with HRP‐conjugated secondary antibody (Invitrogen, Catalog #31460 for rabbit and #31430 for mouse) at room temperature for 1–3 hr. The blots were developed using ImageQuant LAS 4000 (GE Healthcare Life Sciences) with HyGLO™ Chemiluminescent HRP Detection Reagent (Denville Scientific, Swedesboro, NJ, USA; Catalog #E2500). For whole cell lysates, the cells were solubilized with 1% SDS, sonicated, and then subjected to SDS/PAGE analysis.

For peptide pull‐down of GST‐BD fusion proteins, purified and eluted GST‐BD2, GST‐BD4, GST‐BD12, GST‐BD23, GST‐BD34, GST‐BD45, and GST‐BD245 were incubated with H3 and H3K14ac peptides for 1 hr, followed by incubation with streptavidin beads for another hr, and then, the beads were washed and boiled before western blot analysis. The intensity of the protein bands on the western blot images from three independent experiments was measured with NIH imagej software (Bethesda, MD, USA). The ratios of H3K14ac peptide‐associated bands over the H3 peptide‐associated bands were calculated and plotted, and the *P* values were calculated with two‐tailed Student's *t*‐test.

Mono‐nucleosome pull‐down with GST‐BD fusion protein was performed as previously described (Slaughter *et al*., 2018). Briefly, 3 μg purified HeLa mono‐nucleosome (EpiCypher, Durham, NC, USA; Catalog #16‐0009) was incubated with 10 μL bed volume of 300 pmoles pre‐immobilized GST‐BD fusion protein bound to glutathione agarose. The assay was performed in binding buffer (1× PBS, 0.1% NP‐40, 0.5% BSA) for 2 h at 4 °C. Beads were washed with binding buffer and boiled with 2× SDS buffer before western blot analysis.

Antibodies used include Flag, Sigma M2 F1804; PBRM1, Bethyl A301‐591A; Vinculin, Santa Cruz Biotechnology sc‐5573, Dallas, TX, USA; IKKα, Santa Cruz Biotechnology sc‐7182; IKKγ, Santa Cruz Biotechnology sc‐8032; Topo IIβ, Santa Cruz Biotechnology sc‐365421; GST, Cell Signaling Technology, #2625 (Danvers, MA, USA). H3K14ac, GeneTex (Zeeland, MI, USA) (GTX88008); GST, Cell Signaling Technology (2625); histone H3, Bethyl A300‐823A (Montgomery, Texas, USA). For the right panel of Fig. 2A, the PBRM1 antibody was generated with GST‐BD23 fusion protein in rabbits.

### 2.6 Cellular fractionation

HeLa cells were transfected with WT and mutant PBRM1, and, after 36 hr, the cells were scraped and centrifuged for 5 min at 100 ***g***. Cell pellets were resuspended in 500 μL hypotonic buffer (10 mm Tris/HCl, pH 8.0, 1.5 mm MgCl_2_, 10 mm KCl and protease inhibitor cocktail [Pierce, Catalog #88666]), incubated on ice for 15 min, then vortexed for 10 s after adding 25 μL Triton X‐100. After centrifuging for 30 s at 17 800 ***g***, the supernatants were collected as the cytoplasmic fraction. The pellets were resuspended in 100 μL hypertonic buffer (20 mm Tris/HCl pH 8.0, 420 mm KCl, 1.5 mm MgCl_2_, 20% [v/v] glycerol and protease inhibitor cocktail) and vortexed for 10 s. Pellets were incubated on ice for 40 min and vortexed for 15 s every 10 min. Supernatants were collected as the nuclear fraction after centrifugation at 17 800 ***g*** for 10 min. The remaining pellets were sonicated with 1% SDS for 3 × 10 s (10 s on/10 s off) using a probe sonicator, and then, the chromatin part was obtained after centrifuging at 17 800 ***g*** for 10 min.

### 2.7 The generation of 786‐O PBRM1 knockout clones and re‐introduction of wild‐type or mutant PBRM1

sgRNAs (PBRM1‐gRNA‐F: CACCGTGGCAACCTGGTTCACCAT, PBRM1‐gRNA‐R: AAACATGGTGAACCAGGTTGCCC) were designed using Optimized CRISPR Design (crispr.mit.edu) and cloned into pSpCas9n(BB)‐2A‐Puro (PX462) V2.0 as previously described (Ran *et al*., 2013). 786‐O cells were transfected with PX462‐sgPBRM1, and, after 36 hr, cells were selected with puromycin (2.0 μg·mL^−1^) for 3 days. Selected cells were then diluted 1 : 100 and transferred into 15‐cm plates. After ~ 10 days, the single colonies were isolated using cloning cylinders and transferred into 24‐well plates. After about 2 weeks, cells were collected for western blot analysis to assess level of PBRM1 knockout.

The full‐length cDNAs of human PBRM1, PBRM1 in‐frame deletion of N263 (BD2IFD) mutant, and PBRM1 3m mutant was cloned into pLNCX‐GFP vector plasmid. The constructs were transfected into AmphoPack‐293 Cell Line (Clontech, Mountain View, CA, USA; Catalog #631505) to generate retrovirus, and then, 786‐O PBRM1 knockdown cells were transduced to stably express wild‐type or mutant PBRM1. Tumor‐derived PBRM1 mutations were described in Table 1.

**Table 1:** Tumor‐derived mutations

Position (AA)	Mutations (CDS)	Mutations (amino acid)	Mutation ID (COSM)	Mutation type	Tissue
232	c.694A>C	p.T232P	COSM52807	Substitution—missense	ccRCC
233	c.698T>C	p.I233T		Substitution—missense	Renal cell carcinoma (OS‐RC‐2) (Varela *et al*., 2011)
254	c.761T>C	p.L254P	COSM52902	Substitution—missense	Renal cell carcinoma
256	c.766G>A	p.A256T	COSM2854199	Substitution—missense	Adenocarcinoma
263	c.787_789delAAT	p.N263delN	COSM1047100	Deletion—in‐frame	Endometrioid carcinoma

### 2.8 Immunofluorescence

HeLa cells were transfected with wild‐type Flag‐PBRM1 or Flag‐PBRM1–3m and split into eight‐well chamber slides 1 day after transfection. The next day, the cells were fixed in 4% paraformaldehyde for 10 min and permeabilized with 0.5% Triton X‐100 for 15 min, followed by three washes with PBS. The cells were blocked with 5% bovine serum albumin for 60 min and incubated with primary anti‐Flag antibody (Invitrogen, Catalog #MA1‐91878) overnight at 4 °C. The next day, the cells were washed three times with PBS and incubated with fluorescent secondary antibodies (Santa Cruz Biotechnology, sc‐2010) for 1 hr. Nuclei were stained using DAPI (Invitrogen, Catalog #D1306). Labeled cells were observed using a green (488 nm) laser under a confocal microscope (NikonC1 Plus laser; Nikon, Melville, NY, USA) to detect PBRM1 subcellular localization.

### 2.9 Quantitative real‐time PCR

Total RNA was extracted from cells using the RNeasy Plus Mini Kit (Qiagen, Venlo, the Netherlands; Catalog #74136) according to the manufacturer's instructions. RNA concentration was determined using the NanoDrop ND‐1000 system (Thermo Fisher, Waltham, MA, USA). Total RNA (1 μg) was reverse‐transcribed using the First Strand cDNA Synthesis Kit (OriGene, Rockville, MD, USA; Catalog #NP100042). Quantitative real‐time PCR was performed using the LightCycler 480 (Roche, Basel, Switzerland). The primers used for real‐time PCR are listed in Table 2. *GAPDH* was used as an internal control.

**Table 2:** Primers for real‐time PCR

*GAPDH‐F*	TGCACCACCAACTGCTTAGC
*GAPDH‐R*	GGCATGGACTGTGGTCATGAG
*OAS1‐F*	CATCTGTGGGTTCCTGAAGG
*OAS1‐R*	GAGAGGACTGAGGAAGACAAC
*IFI44L‐F*	GAGCACAGAAATAGGCTTCTAGC
*IFI44L‐R*	TGGTATCAGACCCCACTACGG
*ARPC4‐F*	TCAGTCAATGCCCGTG
*ARPC4‐R*	CTCCAGGACGCCTTCG
*PBRM1‐F*	GTGTGATGAACCAAGGAGTGGC
*PBRM1‐R*	GATATGGAGGTGGTGCCTGCTG

### 2.10 ChIP

ChIP assays were performed using the Pierce Agarose ChIP Kit (Thermo Scientific, Waltham, MA, USA; Catalog #26156) according to the manufacturer's instructions. Briefly, samples were cross‐linked with paraformaldehyde (final concentration 1%) and incubated at room temperature for 10 min. The cross‐linking reaction was halted with glycine (final concentration 125 mm) and incubated for 5 min at room temperature. Cells were washed twice with cold 1× PBS and detached by scraping. Cells pellets were suspended in Lysis Buffer 1 containing protease inhibitors and incubated on ice for 10 min. Nuclei were resuspended in 100 μL MNase digestion buffer containing 0.25 μL micrococcal nuclease (10 U·μL^−1^) and incubated at 37 °C for 15 min. The reaction was stopped with 10 μL MNase Stop Solution. Nuclei were pelleted by centrifugation at 9000 ***g*** for 5 min, then resuspended in 50 μL lysis Buffer 2 containing protease/phosphatase inhibitors, and incubated on ice for 15 min. The supernatant, containing the digested chromatin, was collected after centrifuging at 9000 ***g*** for 5 min. Samples were diluted in 1× IP dilution buffer and incubated with PBRM1 antibody (Millipore, Burlington, MA, USA; Catalog #ABE70) overnight at 4 °C. Twenty microliter ChIP Grade Protein A/G Plus Agarose was added into each IP and incubated for 1 hr at 4 °C. The agarose resins were washed once with IP Wash Buffer 1, twice with IP Wash Buffer 2 and once with IP Wash Buffer 3, each with gentle rocking at 4 °C for 5 min. One hundred fifty microliter 1× IP Elution buffer was added to the washed resin and incubated at 65 °C for 30 min with shaking. Six microliter of 5 m NaCl and 2 μL of 20 mg·mL^−1^ Proteinase K were added to each sample and incubated at 65 °C for 1.5 h. Then, each eluted IP was washed with DNA column wash buffer and eluted with 50 μL of DNA Column Elution buffer. The resulting purified DNA was used in real‐time PCR detection using the primers listed in Table 3. *GAPDH* was used as an internal control.

**Table 3:** Promoter‐specific primers for ChIP

*ZNF395‐F*	CAAGTTTGGCTCCGGGCTGC
*ZNF395‐R*	CCTGACCGAGTGGTGACCC
*UBE2L6‐F*	CACGGAGCTGCGTCTGTACA
*UBE2L6‐R*	CGGCCGCACCGGGAGCTCTC
*IGFBP3‐F*	GAGCACTGCGGCTGGGCGCT
*IGFBP3‐R*	GGCACGCTGCTTGGCAGGCT
*GAPDH‐F*	TGAGCCCGCAGCCTCCCGCT
*GAPDH‐R*	CCGGGTTTCTCTCCGCCCGT

### 2.11 Nude mice subcutaneous xenograft assays

The recommendations in the Guide for the Care and Use of Laboratory Animals of the National Institutes of Health were strictly followed to perform all animal experiments. Protocol (01462‐935A) was approved by a Thomas Jefferson University Animal Care and Use Committee and protocol 2015‐11286 approved by the IACUC of Yale University. Subcutaneous xenograft assays in nude mice were performed as previously described (Zhang *et al*., 2013). 786‐O cells at 70–80% confluency were trypsinized and washed twice with ice‐cold PBS. 10^7^ cells in 100 μL PBS were injected subcutaneously into one flank of a nude mouse, and the same number of cells of another cell line was injected into the other flank of the same mouse. The mice were sacrificed 6–10 weeks later, and tumors were excised and weighed.

### 2.12 Statistical analysis

Statistical significance was determined by the two‐tailed unpaired Student's *t*‐test. Tumor weights were assessed in the one‐way ANOVA test using graphpad prism v7.0 (San Diego, CA, USA). In all figures, statistical significance is represented as mean ± SEM, and a *P*‐value ≤ 0.05 was considered statistically significant. *P ≤ 0.05, **P ≤ 0.01, ***P ≤ 0.001, ****P ≤ 0.0001.

## 3 Results

### 3.1 Full‐length PBRM1 has strong and specific binding to H3K14ac

**Figure 1:**
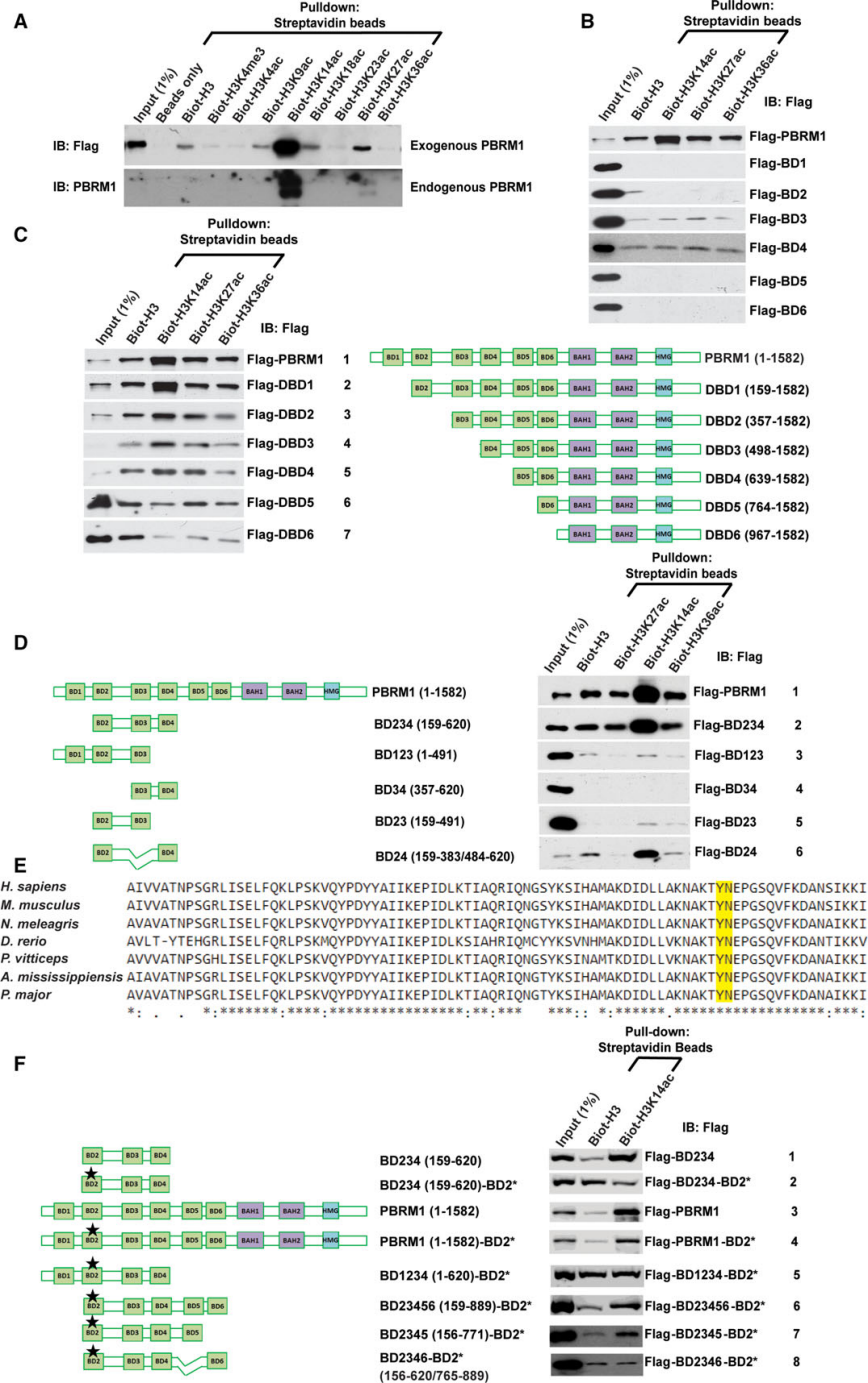
BDs 2, 4, and 5 collaborate to strongly bind H3K14ac. (A) Flag‐PBRM1 was expressed in HEK293T cells. The EBC cell lysates were pulled down with indicated biotinylated peptides and analyzed with indicated antibodies (top). HEK293 lysates were treated the same way to examine the interaction between endogenous PBRM1 and biotinylated histone H3 peptides (bottom). (B) Full‐length PBRM1 or individual BDs of PBRM1 were expressed in HEK293T cells. Their binding to different biotinylated peptides was analyzed as described in (A). (C) Full‐length PBRM1 or PBRM1 with BDs sequentially truncated was expressed in HEK293T cells and analyzed as described above. (D) Full‐length PBRM1 or different combinations of BDs of PBRM1 were expressed in HEK293T cells and analyzed as described above. (E) The aligned protein sequences of BD 2 from different species, and the conserved YN residues that are critical for acetyl‐lysine recognition were labeled with a box. (F) Full‐length PBRM1 or different combinations of BDs of PBRM1, in the presence or absence of indicated mutations, were expressed in HEK293T cells and analyzed as described above. BD2* indicates N263A point mutation in BD2.

We first examined the binding of full‐length PBRM1 to acetylated histone H3 peptides. Nuclear lysates from HEK293 cells were incubated with biotinylated histone H3 peptides that were nonmodified, methylated at K4, or acetylated at K4, K9, K14, K18, K23, K27, or K36. Both exogenous and endogenous full‐length PBRM1 displayed strong binding to the H3K14ac peptide but not to the H3 peptides acetylated on other lysine residues (Fig. 1A). Additionally, PBRM1 did not bind to the nonmodified or K4 trimethylated H3 peptides (Fig. 1). Our results suggest that PBRM1 specifically interacts with H3K14ac.

### 3.2 Bromodomains 2, 4, and 5 collaborate to strongly bind H3K14ac

We next investigated which PBRM1 BD(s) mediate recognition of H3K14ac. Flag‐tagged constructs containing either full‐length PBRM1 or individual BDs plus adjacent sequences on both ends were expressed in HEK293 cells. Nuclear lysates were incubated with biotinylated H3 peptides that were nonmodified or acetylated on K14, K27, or K36. Full‐length Flag‐PBRM1 behaved as expected showing robust binding to the H3K14ac peptide. However, while BD3 and BD4 showed modest binding to the peptides, none of the individual BD constructs displayed the same level of binding toward H3K14ac compared to full‐length PBRM1 (Fig. 1B), suggesting that cooperation between multiple BDs may be required for high affinity binding to H3K14ac. To address this possibility, PBRM1 constructs harboring deletions of the BDs were generated, beginning with deletion of BD1 at the N terminus and containing successive deletions of each BD (DBD1–6, Fig. 1C). Peptide pull‐down assays were conducted with nonmodified or acetylated H3 peptides. Flag‐DBD6, which lacks all the BDs of PBRM1, showed negligible binding to H3K14ac. While deletion of BD1 (Flag‐DBD1, row 2) did not affect H3K14ac binding in this assay, deletion of BDs 1 and 2 (Flag‐DBD2, row 3) greatly reduced the binding of PBRM1 to H3K14ac. Deletion of BDs 1–4 (Flag‐DBD4, row 5) further reduced binding to H3K14ac compared to deletion of BDs 1–3 (Flag‐DBD3, row 4). Further deletion of BD5 (Flag‐DBD5, row 6) completely abolished binding of H3K14ac versus the nonacetylated H3 peptide (Fig. 1C), suggesting that BDs 2, 4, and 5 contribute to H3K14ac binding.

Since the sequential deletion of BDs only reduced PBRM1's affinity to H3K14ac gradually, we used different combinations of BDs 1–4 to critically examine their contributions to H3K14ac binding. The combination of BDs 2, 3, and 4 (Flag‐BD234, Fig. 1D, row 2) showed a similar level of binding toward H3K14ac as full‐length PBRM1 (row 1), while the construct containing BDs 1, 2, and 3 (Flag‐BD123) showed no affinity to H3K14ac (Fig. 1D, row 3), indicating that BD4 is important for the interaction. Additionally, the construct containing BDs 2 and 4 (Flag‐BD24, row 6), but not 2 and 3 or 3 and 4 (Flag‐BD23 and Flag‐BD34, rows 4 and 5 respectively), showed high affinity to H3K14ac (Fig. 1D). Altogether, results show that the combination of BDs 2 and 4 of PBRM1 creates high affinity to H3K14ac, while BDs 1 and 3 contribute significantly less to the binding.

The tyrosine‐rich binding pocket in BD 2 contains a YN sequence that is highly conserved through evolution (Fig. 1E, top panel). The asparagine at position 263 (N263) had been previously found to be critical for the recognition of acetyl‐lysine by forming a hydrogen bond with the acetylated lysine on target proteins (Dhalluin *et al*., 1999; Owen *et al*., 2000). Here, the N263A mutation (denoted as BD2*), which disrupts the YN motif in BD2, abolished the enhanced affinity of Flag‐BD234‐BD2* toward H3K14ac over the nonacetylated peptide (Fig. 1F, row 2). BD2* also reduced H3K14ac binding by full‐length PBRM1 in Flag‐PBRM1‐BD2* (compare rows 3 and 4), but the enhanced binding to H3K14ac over nonacetylated peptide still persisted, suggesting that BDs other than BD2 are also critical for the interaction. Confirming the result in Fig. 1D, the addition of BD1 did not help Flag‐BD234‐BD2* to bind H3K14ac (Fig. 1F, row 5). Instead, the addition of BDs 5 and 6 did (Fig. 1F, row 6). Leaving out BD6 did not alter H3K14ac binding, while in‐frame deletion of BD5 in Flag‐BD2346‐BD2* abolished the recognition of H3K14ac over nonacetylated peptide (Fig. 1F, rows 7 and 8). This suggests that BD5 of PBRM1 also makes a critical contribution to H3K14ac recognition.

### 3.3 *In vitro* measurement confirms that BDs 2, 4, and 5 collectively bind H3K14ac with high affinity

**Figure 2:**
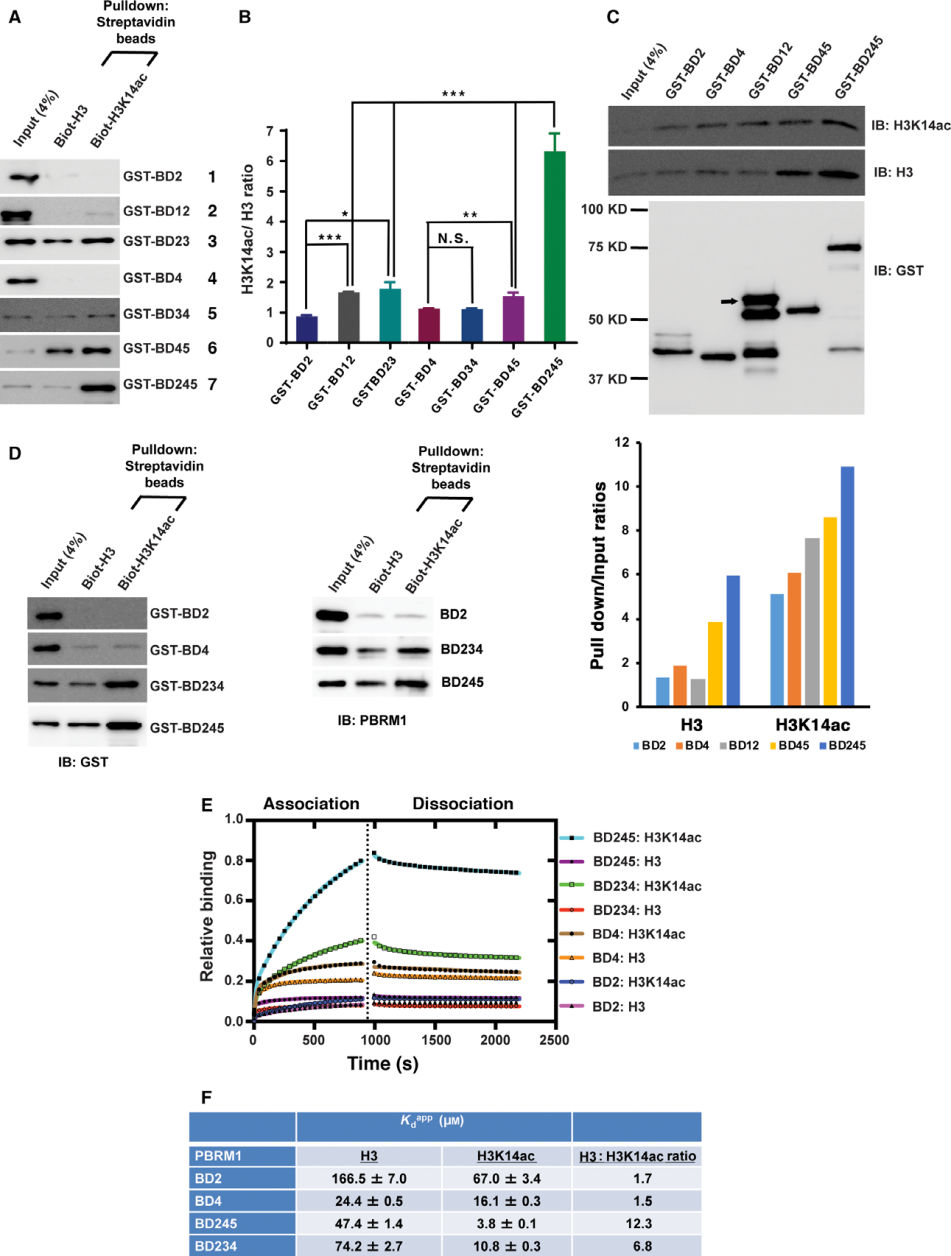
Quantitative measurement confirms that BD245 work together to create high affinity to H3K14ac. (A) The indicated GST‐BD constructs of PBRM1 were expressed and purified from *Escherichia coli*, pulled down by the indicated peptides, and immunoblotted with anti‐GST antibody. (B) The bands in (A) were quantified with NIH ImageJ, and the ratios of bands associated with H3K14ac/H3 were calculated from three experiments. The *P*‐values were calculated using the two‐tailed Student's *t*‐test. *: *P* < 0.05; **: *P* < 0.01; ***: *P* < 0.001. N.S.: nonsignificant. (C) Top: The indicated GST‐BD constructs of PBRM1 were expressed and purified from *Escherichia coli*. Similar amount of GST‐fusion protein was incubated with 3ug of nucleosome purified from HeLa cells, washed then analyzed with indicated antibodies. Arrow: the full‐length GST‐BD12 protein. Bottom: The intensity of the pulled down H3 or H3K14ac bands was determined with ImageJ from three different experiments, and the ratios of H3/input and H3K14ac/input were calculated and plotted. The error bars represent standard error of the mean. (D) GST‐BD fusion proteins (left) or cleaved and purified BD proteins (right) were pulled down with indicated peptides and analyzed with western blots. (E) BLI analysis of the interaction between PBRM1 BD proteins and the H3K14ac peptide. Binding activity was normalized to maximum response and is reported as relative binding. The association phase takes place from 0 to 900 s; the dissociation phase takes place from 901 to 2200 s. (F) Binding parameters of PBRM1 BD constructs and H3 peptide interactions. Apparent *K*_d_ values ($K_{d}^{\mathrm{app}}$) were obtained for the interaction between PBRM1 BD proteins and H3 peptides by fitting BLI data to a double exponential function. The H3:H3K14ac reports the preference of PBRM1 BD proteins to bind the H3K14ac peptide over the nonacetylated H3 peptide.

To examine the conclusions made by Flag‐BD constructs, we generated GST‐BD constructs and tested their binding to histone H3 peptides. GST‐BD2 or 4 again showed little binding under the stringent binding condition. GST‐BD12 showed weak binding to H3K14ac (Fig. 2A row 2 versus 1, 2B), suggesting that BD1 helps BD2 to bind it, consistent with a previous report (Porter and Dykhuizen, 2017). BD3 helped BD2, but not much so for BD4, to bind H3K14ac, likely by increasing the affinity to the nonacetylated peptide backbone (Fig. 2A row 3 versus 1 and row 5 versus 4, 2B). BD5 also helped BD4 to bind H3K14ac, consistent with the Flag‐BD result (Figs 2A row 6 versus 4, 2B and 1F). BD245 displayed the strongest and specific binding to H3K14ac compared to other strong binders (Fig. 2A row 7 versus the rest and 2B). The conclusions on BD 1 and 3 are somewhat different from those derived from Flag‐BD pull‐downs, suggesting that subtle differences can be revealed if the condition is permissive.

To confirm that our conclusions are not artifacts created by binding to peptides, we incubated GST‐BD proteins with mono‐nucleosome purified from HeLa cells. GST‐BD2, BD4, BD12, or BD45 all pulled down histone H3 and enriched H3K14ac, but again BD245 showed the strongest binding to histone H3 and H3K14ac (Fig. 2C top, lane 6 versus the rest). GST‐BD12 had some obvious breakdown products and they might interfere with binding to the nucleosome. After quantification, it was found that all BD constructs had higher ratios of H3K14ac/input over H3/input, suggesting that these constructs demonstrated selective binding to the modification (Fig. 2C, bottom). The result suggests that BD245 has the highest affinity to H3K14ac even when it is present in intact nucleosome.

To confirm the roles of BDs 2, 4, and 5 in recognition of H3K14ac, we measured the interactions between histone H3 peptides and purified BD proteins. GST‐fusion proteins of BD2, BD4, BD234, and BD245 were purified, and their ability to bind biotinylated histone H3 peptides was assessed. The peptide pull‐down assay showed little to no interaction between GST‐BD2 and GST‐BD4 with H3K14ac, while GST‐BD234 and GST‐BD245 had significantly higher affinity toward H3K14ac than to the nonacetylated H3 peptide (Fig. 2D, left panel). After GST‐tag removal, the BD proteins still retained their previous characteristics and were used for further analysis (Fig. S1 and Fig. 2D, right panel).

To validate the detected interactions, we compared binding among the BD proteins using BLI. Figure 2E shows the results of the BLI assay measuring the interaction of a similar concentration of each BD construct on sensors loaded with biotinylated histone H3 or H3K14ac peptides. BD2, BD4, BD234, and BD245 all showed histone H3‐dependent association with sensors, albeit with distinctly different binding levels and rates of association. BD245 and BD234 showed distinct preferences for binding acetylated versus nonacetylated H3K14 peptides with binding ratios of 12.3 and 6.8, respectively (Fig. 2F). In contrast, BD2 and BD4 domains showed little preference for acetylated versus nonacetylated H3K14 peptides (binding ratios of 1.7 and 1.5, respectively; Fig. 2F). Little difference in dissociation rates was observed when bound sensors were incubated in buffer (right side of dotted line in Fig. 2E), suggesting that the differences in affinity were largely due to the different rates of association. In addition, the data for BD245 and BD234 were best fit using a double exponential rate equation, suggesting that high affinity may be achieved by real multivalent or artifactual surface interactions. Given that the trends in affinities match those observed in peptide pull‐down assays, real multivalent interactions are more likely. Taken together, quantitative measurements by BLI confirmed the observation that BDs 2, 4, and 5 contribute to high affinity binding of H3K14ac by PBRM1.

### 3.4 Concurrent point mutations in BDs 2, 4, and 5 abolish PBRM1's recognition of H3K14ac and alter its subcellular localization

**Figure 3:**
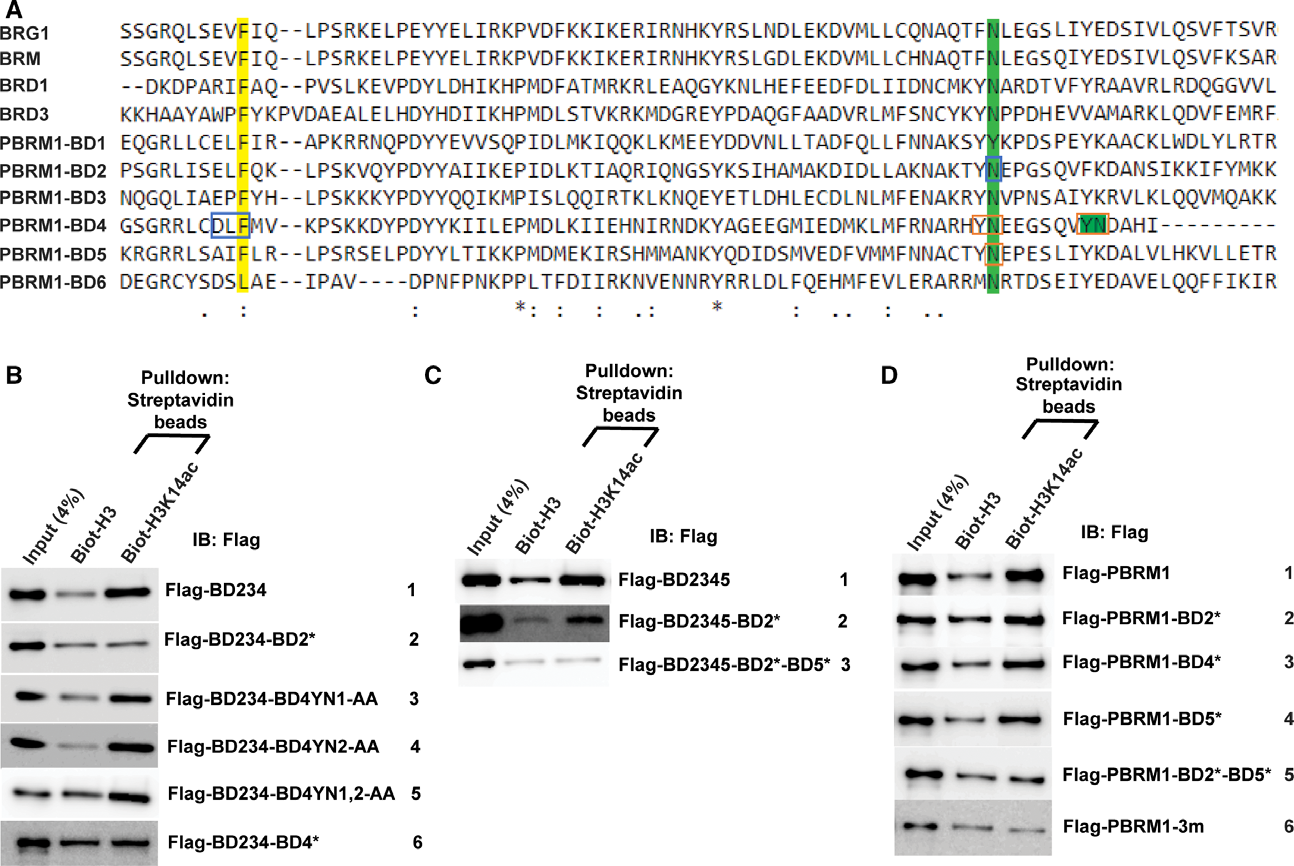
Concurrent point mutations in BDs 2, 4, and 5 abrogate PBRM1's recognition of H3K14ac. A) Sequence alignment of the six BDs of human PBRM1 and the BDs of BRG1 and BRM. *: conserved residues : or.: highly similar amino acids. The residues mutated in B–D were boxed. (B, C, and D) The indicated PBRM1 constructs were expressed in HEK293T cells, pulled down by the indicated peptides, and immunoblotted with anti‐Flag antibody. The * in the rows indicate point mutations.

Previous results demonstrated that the conserved YN residues in BD2 are critical for H3K14ac binding (Fig. 1E) (Chandrasekaran and Thompson, 2007; Charlop‐Powers *et al*., 2010; Porter and Dykhuizen, 2017). We next tested whether the conserved YN residues in BD4 and/or BD5 are also critical for H3K14ac binding. In addition to the highly conserved YN motif in BD4 (residues: 600–601), there is a second, less‐conserved YN motif (residues: 608–609) in BD4. These motifs are disrupted by the N601A (YN1) and N609A (YN2) mutations (Fig. 3A). Neither of the single mutations nor the double mutation changed the affinity of BD234 toward H3K14ac (Fig. 3B, rows 3, 4 and 5), suggesting BD4 uses a different mechanism to achieve H3K14ac recognition. We aligned the amino acid sequences of BDs from human PBRM1, BRG1, and BRM and identified a DLF sequence in BD 4, which is highly conserved among BD4 of PBRM1 from different species and has similar amino acid residues in different BD domains (Fig. 3A). We mutated these residues to alanine (DLF>AAA, BD4*) and found that it greatly reduced binding of BD234 to H3K14ac (Fig. 3B, row 6), indicating that this highly conserved sequence in BD4 is required for H3K14ac binding. Simultaneous mutation of the YN motifs in BDs 2 and 5 (N263A and N739A, BD2* and BD5*) abolished the affinity of BD2345 toward H3K14ac compared to the N263A single mutation (Fig. 3C, compare rows 2 and 3), confirming that the YN motif in BD5 is also critical for H3K14ac binding. Altogether, results show that the YN motifs of BDs 2 and 5 as well as the DLF sequence of BD4 are necessary for H3K14ac recognition.

A recent publication suggested that a ccRCC‐derived BD4 N601K mutation could significantly reduce H3K14ac recognition in GST‐BD4 and BD45 (Slaughter *et al*., 2018). We compared it with Y600N601‐AA mutant and found that it did significantly reduce the binding to H3K14ac in Flag‐BD234 (Fig. S2). Thus, this N601K mutation might change the structure of BD4 while the Y600N601‐AA may not.

After defining the critical binding residues of BDs 2, 4, and 5 with smaller constructs, we investigated how mutation of these residues affects the binding between full‐length PBRM1 and H3K14ac. The individual N263A (BD2*), DLF>AAA (BD4*), and N739A (BD5*) mutations each slightly reduced the affinity toward H3K14ac (Fig. 3D, rows 2, 3, and 4), while the N263A‐N739A (BD2* and 5*) double mutation reduced the affinity further (Fig. 3D, row 5). The triple mutation N263A‐DLF>AAA‐N739A (3m) completely abolished the ability of full‐length PBRM1 to bind H3K14ac (Fig. 3D, row 6), indicating that concurrent mutations within BDs 2, 4, and 5 are necessary and sufficient to prevent specific recognition of H3K14ac.

**Figure 4:**
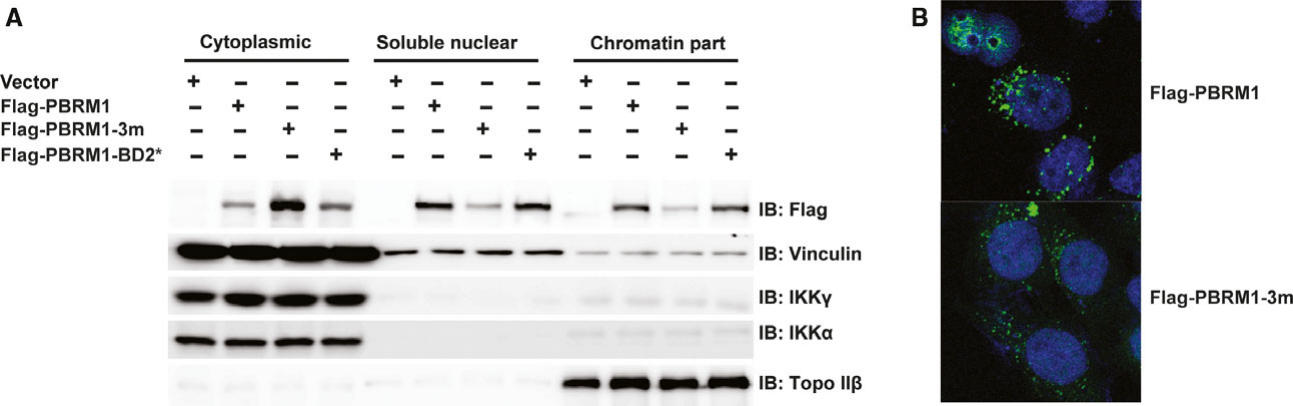
Concurrent point mutations in BDs 2, 4, and 5 cause PBRM1 relocalization to the cytoplasm, while mutation in BD2 alone does not. (A) Wild‐type or mutant PBRM1 constructs were transiently expressed in HEK293T cells. The cells were fractionated into cytoplasmic, soluble nuclear, and chromatin‐bound fractions. The lysates were blotted with the indicated antibodies. The blots of IKKγ, IKKα, and Topo IIβ indicate the successful separation of cellular compartments. (B) Wild‐type or mutant PBRM1 constructs were expressed in HeLa cells. Immunofluorescence was performed using anti‐Flag antibody. The DNA is stained blue with DAPI, while the PBRM1 signal is stained green.

Polybromo‐1 is a chromatin‐associated protein (Porter and Dykhuizen, 2017). To examine the influence of BD mutations on PBRM1 localization, we transfected HeLa cells with wild‐type, the triple BD mutant, or the BD2 mutant. Cells were then fractionated into the cytoplasmic, soluble nuclear, and chromatin‐bound fractions. Wild‐type PBRM1 was present in each of the cellular compartments but was predominantly localized to the nucleus (Fig. 4A). The triple BD mutant, which is unable to recognize H3K14ac, accumulated in the cytoplasm, with low levels found in the soluble nuclear and chromatin‐bound fractions (Fig. 4A). In contrast, PBRM1 with a mutation only in BD2 had a similar distribution to the wild‐type protein (Fig. 4A). Immunofluorescence confirmed the findings, as wild‐type PBRM1 mainly localized in the nucleus and the triple BD mutant primarily localized to the cytoplasm (Fig. 4B and Fig. S3). This analysis suggests that loss of H3K14ac recognition alters the subcellular localization of PBRM1.

### 3.5 Tumor‐derived mutations in BD2 result in loss of H3K14ac binding, loss of promoter binding, and deregulation of gene expression

**Figure 5:**
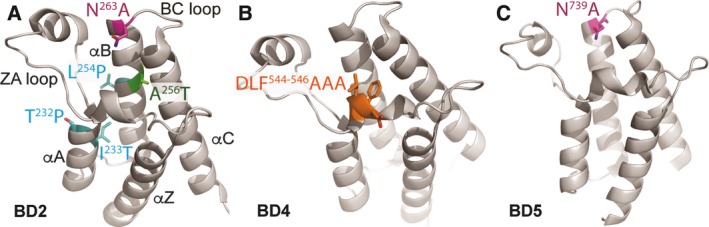
Tumor‐derived mutations mapped onto crystal structures of BDs. (A) Tumor‐derived mutations were mapped onto the available crystal structure of BD2 of PBRM1 (Filippakopoulos *et al*., 2010). (B) BD4* mutation was mapped onto the available crystal structure of BD4 of PBRM1 (Filippakopoulos *et al*., 2010). (C) The location of the critical N739 was mapped onto the available crystal structure of BD5 of PBRM1 (Filippakopoulos *et al*., 2010).

It has been reported that ~ 40% of ccRCC harbor inactivating mutations in PBRM1 (Varela *et al*., 2011). Other cancers also have PBRM1 mutations. Many missense mutations have been found in the BDs. Mapping the tumor‐derived mutations to the available crystal structures of the PBRM1 BDs 2, 4, and 5 (Filippakopoulos *et al*., 2010) provides further insight into the mechanism of how these mutations described in Table 1 may affect PBRM1 function. Mutation N263A changes the highly conserved asparagine residue at the C‐terminal end of helix αB in BD2 (Fig. 5A), thereby removing a critical hydrogen bond between the asparagine and the acetyl‐lysine moiety, which often abolishes binding of acetyl‐lysine peptides (Dhalluin *et al*., 1999). Since I233 belongs to a group of highly conserved hydrophobic residues in helix αA that contribute to the stability of the BD core (Filippakopoulos *et al*., 2010), its mutation to T in BD2 (Fig. 5A) is likely to destabilize the BD fold. Similarly, mutation of A256 in helix αC to T is predicted to interfere with the tightly packed hydrophobic core of BD2 (Fig. 5A), leading to destabilization. While residues T232 and L254 occur in less‐conserved positions and hence do not seem to adopt a specific structural role in BDs, their mutations to prolines may interfere with the BD fold, as prolines introduce kinks into α‐helices. In summary, most tumor‐derived mutations in PBRM1 BD2 structurally and functionally affect important conserved residues, leading to detrimental effects either on acetyl‐lysine peptide recognition or BD stability. The DLF segment mutated to AAA in BD4 resides in a short helical turn with a highly conserved phenylalanine (Fig. 5B) that is thought to stabilize the hydrophobic binding pocket through interactions with helix αC (Filippakopoulos *et al*., 2010). N739A also changes the highly conserved asparagine residue at the C‐terminal end of helix αB in BD5 (Fig. 5C).

**Figure 6:**
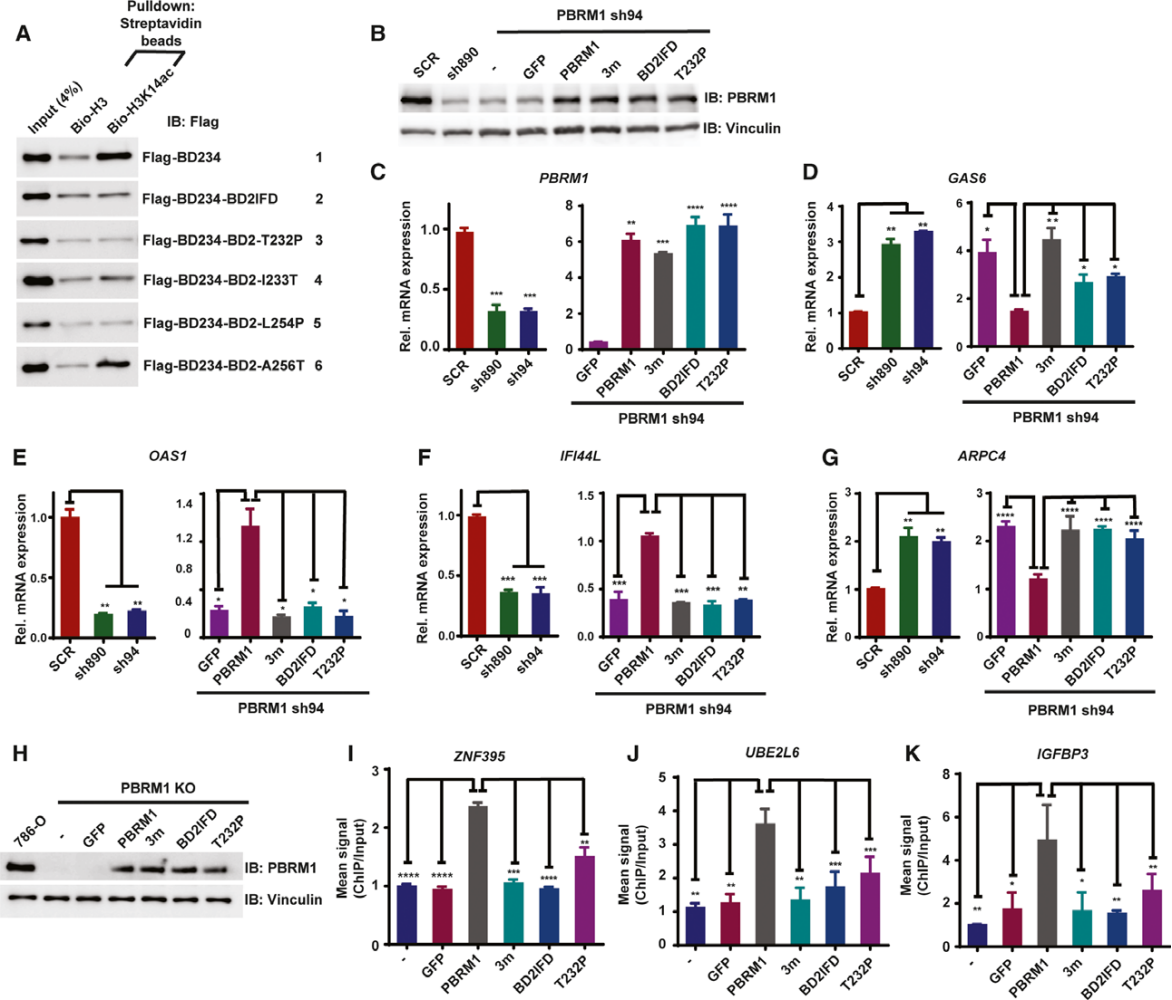
Tumor‐derived mutations in BD2 weaken PBRM1's recognition of H3K14ac, its ability to regulate gene expression, and its binding to promoters. (A) Wild‐type BD234 of PBRM1 or BD234 carrying tumor‐derived mutations were expressed in HEK293T cells. The lysates were pulled down with the indicated peptides and immunoblotted with anti‐Flag antibody. (B) 786‐O cells were infected to stably express the indicated shRNA and/or PBRM1 constructs. The lysates were blotted with indicated antibodies. The relative mRNA expression of (C) *PBRM1*, (D) *GAS6*, (E) *OAS1*, (F) *IFI44L,* and (G) *ARPC4* over SCR‐expressing cells was examined with qRT–PCR in the indicated cells from two experimental results. (H) PBRM1 constructs or GFP was transiently transfected into 786‐O cells with PBRM1 knocked out by CRISPR/Cas9. Anti‐PBRM1 ChIP was performed and the enrichment of promoter sequences for (I) ZNF‐395, (J) UBE2L6, or (K) IGFBP3 was measured by qPCR. The ChIP/input ratio for each promoter from the nontransfected cells was set as 1, and the relative enrichment was calculated by dividing the ChIP/input ratio from the transfected cells with that from the control cells from two experimental results. The error bars represent standard error of the mean. The *P*‐values were calculated using the two‐tailed Student's *t*‐test. *: *P* < 0.05; **: *P* < 0.01; ***: *P* < 0.001; ****: *P* < 0.0001.

We hypothesized that these tumor‐derived mutations would alter PBRM1's function through impairing its ability to recognize H3K14ac. We used site‐directed mutagenesis to introduce tumor‐derived mutations into Flag‐BD234. The N263A mutation abolished H3K14ac recognition in BD234 but only reduced it in BD2345 (Fig. 3B,C, compare rows 1 and 2), so Flag‐BD234 is more sensitive to the perturbation of H3K14ac recognition by mutations in BD2. The BD2IFD, T232P, and L254P mutations completely abolished H3K14ac binding (Fig. 6A, rows 2, 3, and 5), while I233T reduced binding and A256T had no apparent effect (Fig. 6A, rows 4 and 6). These mutations all occur in BD2. These results suggest that many tumor‐derived mutations in PBRM1's BD2 impair its ability to recognize H3K14ac, indicating that this binding event may be critical for its tumor suppressor function.

Histone lysine acetylation is important during transcription and is generally linked to gene activation (Wang *et al*., 2008). We predicted that BD mutations that disrupted H3K14ac binding would negatively impact PBRM1's role in gene expression. We employed microarray analyses to profile differentially expressed genes upon PBRM1 shRNA knockdown in 786‐O ccRCC cells. Knockdown of PBRM1 led to upregulation of *GAS6* and *ARPC4*, and downregulation of *OAS1* and *IFI44L* relative to control scrambled shRNA cells. Re‐expression of shRNA‐resistant wild‐type PBRM1 or PBRM1 BD mutants (Flag‐PBRM1–3m, Flag‐PBRM1‐BD2IFD, Flag‐PBRM1‐T232P) in PBRM1 knockdown cells restored PBRM1 protein similar to endogenous levels and increased PBRM1 mRNA levels in 786‐O cells (Fig. 6B,C). Expression of wild‐type PBRM1 reversed the effects of PBRM1 knockdown, leading to downregulation of *GAS6* (Fig. 6D), upregulation of *OAS1* (Fig. 6E) and *IF144L* (Fig. 6F), and downregulation of *ARPC4* (Fig. 6G). Neither the triple BD mutant nor the BD2 mutants harboring BD2IFD or T232P were able to rescue the defects in gene expression resulting from PBRM1 suppression (Fig. 6D–G). Taken together, our data suggest that BD mutations that weaken H3K14ac recognition impair the ability of PBRM1 to regulate gene expression.

We carried out ChIP followed by sequencing to identify PBRM1 target promoters and detected *ZNF395*,* UBE2L6,* and *IGFBP3* promoters among them. To determine whether the BD mutations affect chromatin binding by PBRM1, we performed ChIP‐qPCR in 786‐O cells with *PBRM1* knocked out by CRISPR/Cas9. GFP, wild‐type PBRM1, PBRM1–3m, and tumor‐derived mutants PBRM1‐BD2IFD and PBRM1‐T232P were transiently expressed in the cells (Fig. 6H). After overexpression of wild‐type PBRM1, ChIP with anti‐PBRM1 confirmed that PBRM1 bound to the promoters of *ZNF395*,* UBE2L6,* and *IGFBP3* (Fig. 6I–K). However, despite equal expression levels as the wild‐type PBRM1, the PBRM1–3m, PBRM1‐BD2IFD, and PBRM1‐T232P mutants demonstrated significantly reduced binding to the promoters (Fig. 6I–K), suggesting that reduced H3K14ac recognition is sufficient to impair the recruitment of PBRM1 to select promoters.

### 3.6 H3K14ac recognition is critical to PBRM1's tumor suppressor function

**Figure 7:**
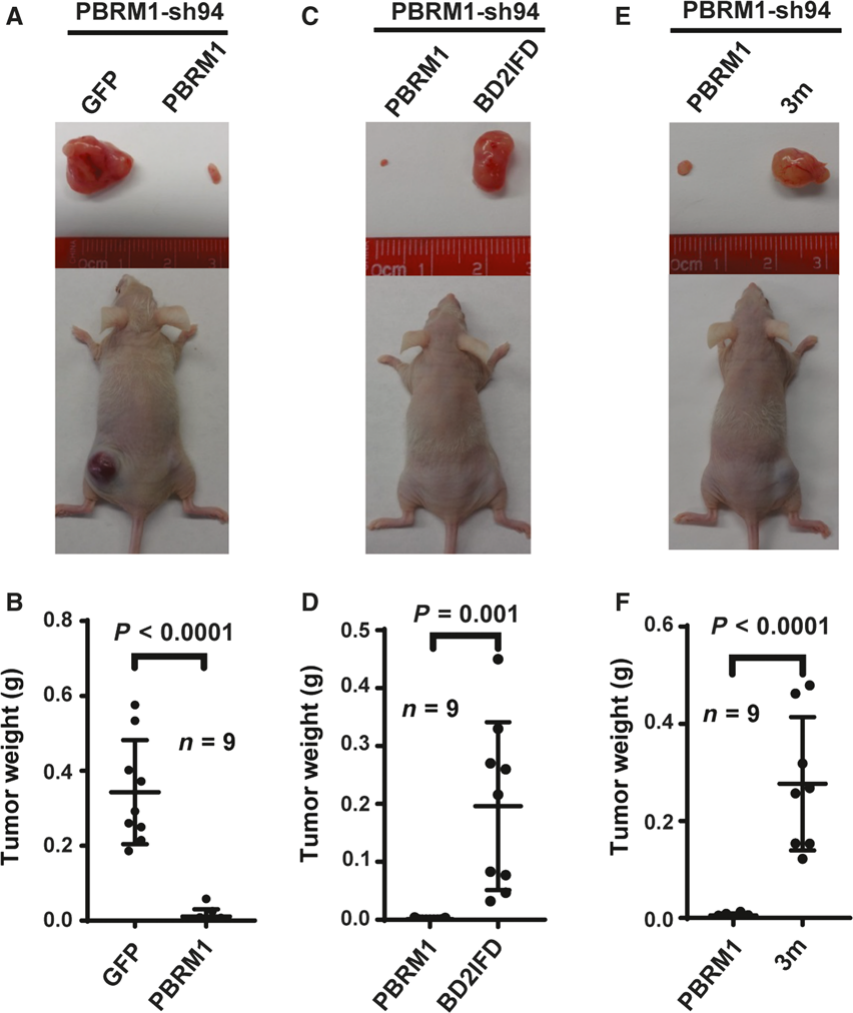
H3K14ac recognition is critical to PBRM1's tumor suppressor function. Xenograft analyses were performed to compare tumor growth of 786‐O cells with PBRM1 stably suppressed by PBRM1‐sh94. (A) Representative tumors derived from cells expressing GFP or shRNA‐resistant wild‐type PBRM1; (B) quantification of tumor weights in A); C) representative tumors derived from cells expressing shRNA‐resistant wild‐type PBRM1 or PBRM1 with N263 in‐frame deletion; (D) quantification of tumor weights in (C); (E) representative tumors derived from cells expressing shRNA‐resistant wild‐type PBRM1 or PBRM1 with mutations in BDs 2, 4, and 5; (F) quantification of tumor weights in (E). The error bar depicts standard error of the mean, and *P*‐values were calculated using the two‐tailed Student's *t*‐test. *n* indicates the number of mice used in each experiment.

Select BD mutations impaired PBRM1's ability to recognize H3K14ac, regulate gene expression, and localize to promoters. Using mouse xenograft models, we tested whether the PBRM1 BD mutations abrogate its tumor suppressor function. We found that 786‐O PBRM1 knockdown cells generated significantly larger tumors than control cells, consistent with previous reports suggesting that PBRM1 is a potent tumor suppressor that inhibits tumor growth (Gao *et al*., 2017). Re‐expression of shRNA‐resistant wild‐type PBRM1 in the knockdown cells resulted in significantly smaller tumors than the GFP‐expressing control (Fig. 7A,B). Overexpression of the tumor‐derived BD2IFD mutant or the 3m mutant failed to significantly suppress tumor growth compared to wild‐type PBRM1 (Fig. 7C–F). These results demonstrate that PBRM1 BD mutants lose tumor suppression function in mouse xenografts, suggesting that H3K14ac recognition is key to PBRM1's tumor suppressor function *in vivo*.

## 4 Discussion

Using mutations in individual BDs in the context of full‐length PBRM1, Porter and Dykhuizen (2017) found that the multi‐BD organization contributed to PBRM1 association with chromatin. They hypothesized that BD2 binds H3K14ac while other BDs bind additional acetylation sites on histone tails, and collectively, these interactions generate high affinity to chromatin. However, it is not clear whether interactions between additional histone acetylation sites and the BDs of PBRM1 play an important role in targeting PBRM1 to the chromatin.

Our work suggests that H3K14ac is a major acetylation mark on histone H3 that recruits PBRM1 to chromatin. Full‐length PBRM1 displayed significantly enhanced affinity toward H3K14ac compared to the nonacetylated counterpart while the individual BDs did not. This observation prompted us to hypothesize that multiple BDs in PBRM1 collaborate to create high affinity to H3K14ac.

The peptide pull‐down assay of PBRM1 in cellular lysates is semiquantitative in nature. In order to quantitatively measure the affinity between H3K14ac and the BDs of PBRM1, either alone or in combination, we produced and purified BD2, BD4, BD234, and BD245 proteins in *E. coli*. With these reagents, we successfully measured the binding efficiency between the proteins and the acetylated and nonacetylated H3 peptides. The results unequivocally confirmed that BD245 and BD234 had significantly higher affinity toward H3K14ac than to its nonacetylated counterpart, while BD2 and BD4 did not show significant preference (Fig. 2C). However, the exact molecular mechanism of this cooperation remains a mystery. One scenario is that one BD binds to H3K14ac while the other BDs bind to nearby residues on the same peptide that may not necessarily include acetylated lysine residues. Although such BD–peptide interactions may be weak, they may provide an avidity effect to generate strong binding. Alternatively, different BDs on one PBRM1 molecule may simultaneously bind to H3K14ac on different molecules that are in close proximity. Since each nucleosome contains two H3, it is possible that two adjacent tails with H3K14ac might provide the binding sites for PBRM1. These hypotheses can be further tested with in‐depth structural analysis with H3K14ac peptide bound to multi‐BD proteins.

Our results are largely consistent with a recent publication in which BDs 2 and 4 are found to be critical for H3K14ac recognition and nucleosome binding (Slaughter *et al*., 2018), and the neighboring BDs modulate the interaction with histone H3 peptides. However, we found that BD245 had the highest affinity to H3K14ac which is critical to anchor PBRM1 in the nucleus. In addition, more subtle mutation of Y600N601‐AA in BD4 did not affect BD4's ability to bind to H3K14ac even though N601K did, suggesting that N601K mutation might disturb the structure of BD4 like the DLF‐AAA mutation.

Here, we also identified point mutations in BDs 2, 4, and 5 that were critical for H3K14ac recognition. Interestingly, BD4 is peculiar in that the conserved N601, a residue that is predicted to be critical to recognize acetyl‐lysine, appears to not be required for H3K14ac recognition. Instead, the conserved DLF motif, which is thought to be critical for the structural integrity of BD4's acetyl‐lysine binding pocket, was required for H3K14ac recognition. Thus, BD4's contribution to H3K14ac recognition is likely not based on its ability to directly bind to acetyl‐lysine. It is possible that BD4 positions or stabilizes BD2 and/or BD5 in a state that yields high affinity for H3K14ac. With N263A (BD2*), DLF>AAA (BD4*) and N739A (BD5*) integrated into full‐length PBRM1 (singly or combined) we found that a point mutation in a single BD only moderately reduced PBRM1's binding to H3K14ac. Only when all three BDs were disrupted simultaneously was the specific binding completely abolished. The triple mutant also lost the affinity to chromatin and relocated to the cytoplasm, while the single BD2* mutant did not change cellular localization (Fig. 4), suggesting that strong H3K14ac recognition is critical to tether PBRM1 to chromatin.

We further tested the impact of tumor‐derived point mutations in BD2. As expected, most of them abolished H3K14ac binding in the BD234 construct. Mapping these mutations onto available crystal structures of the PBRM1 BDs revealed that these mutations impact either important structural or functional residues. Interestingly, the individual point mutants were nearly as defective as the triple mutant in regulating gene expression, binding to promoters (Fig. 6B–K), and suppressing tumor growth (Fig. 7). This suggests that even though tumor‐derived point mutations in the BDs of PBRM1 might not grossly alter chromatin association, they are sufficient for the disruption of PBRM1's molecular and tumor suppressor functions.

Porter and Dykhuizen (2017) and Slaughter *et al*. (2018) used *in vitro* growth curves of Caki‐2 or RCC4 cells re‐expressing wild‐type or BD mutants as a gauge of PBRM1's tumor suppressor function. However, this might not be an accurate measure. In PBRM1‐proficient 786‐O cells, deletion or suppression of PBRM1 did not alter cell growth in culture, but it significantly enhanced tumor growth *in vivo* (Gao *et al*., 2017). Thus, we think that xenograft analysis is a better tool to assess PBRM1's tumor suppressor function than measuring proliferation in cell culture. The knock‐in of *Pbrm1* mutants into the mouse genome combined with *Vhl* inactivation should be the best physiological model to evaluate the impact of these mutations.

## 5 Conclusions

It has long been a puzzle: Why does PBRM1 have six tandem BDs? Our analysis provides a clear answer to this question: Individual BDs of PBRM1 does not have high enough affinity to H3K14ac to allow proper binding. Instead, BDs 2, 4, and 5 work together to generate high affinity toward H3K14ac. If this recognition is disrupted by simultaneous mutation of all three BDs, PBRM1 will relocalize to the cytoplasm. However, a single point mutation in BD2 alone that reduces H3K14ac recognition was sufficient to severely impair PBRM1's molecular and tumor suppressor functions despite maintaining chromatin association. So our findings suggest that PBRM1's molecular and tumor suppressor functions depend on the recognition of H3K14ac by multiple BDs, and PBRM1 is an important reader of H3K14ac, a universal epigenetic marker of actively transcribing genes. Many tumor‐derived mutations disrupt this recognition, attesting to its importance. As PBRM1 deficiency has been shown to boost the efficacy of immune checkpoint drugs in ccRCC and other cancers (Miao *et al*., 2018; Pan *et al*., 2018), the disruption of H3K14ac recognition by PBRM1 has the potential to increase the response rate of immunotherapy and warrants further investigation.

## Author contributions

LLi, NLAV, XHN, WJC, EAC, CBG, and MLZ performed the experiments. MSC contributed to writing of the manuscript. LLa and QY assisted with editing. MSC and HFY designed the experiments and oversaw the execution. LLi, NLAV prepared the figures. QY, MSC, and HFY provided resources to carry out the experiments. HFY wrote the manuscript.

## Conflict of interests

The authors declare no conflict of interests.

## Supporting information


**Fig. S1**. The production of purified BD proteins. GST‐BD fusion proteins were induced in E. coli and the purified proteins were cleaved with TEV protease. The lysates and proteins were resolved by SDS‐PAGE and stained with coomassie blue. Red arrow: BD proteins. Green arrow: GST protein.

**Fig. S2**. N601K mutation in BD4 reduces H3K14ac binding while Y600N601‐AA mutation does not. The indicated Flag‐BD234 constructs were expressed in HEK293T cells, pulled down by the indicated peptides, and immunoblotted with anti‐Flag antibody.

**Fig. S3**. Concurrent point mutations in BDs 2, 4 and 5 cause PBRM1 to relocalize to the cytoplasm. Flag‐tagged wild‐type or 3m mutant PBRM1 constructs were expressed in HeLa cells. Immunofluorescence was performed using anti‐Flag antibody. The DNA is stained blue with DAPI, while the PBRM1 signal is stained green.

## Data Availability

All data are included in the manuscript. All the materials are available upon request.
