# Supplementary figures and images for: High affinity binding of H3K14ac through collaboration of bromodomains 2, 4 and 5 is critical for the molecular and tumor suppressor functions of PBRM1
Source: Mol Oncol. 2019 Feb 2;13(4):811–28. doi: 10.1002/1878-0261.12434 (PMC6441893; doi:10.1002/1878-0261.12434)

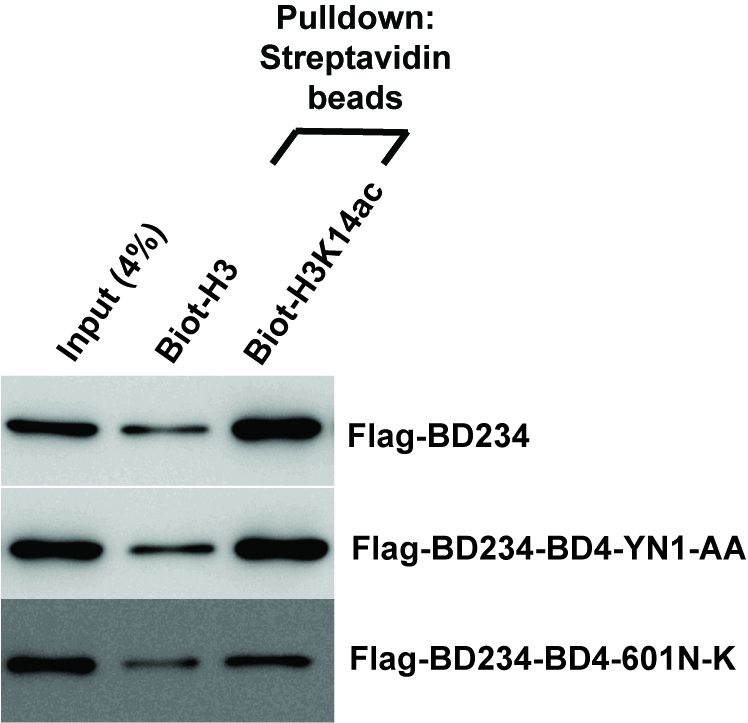

Supplement: Supplementary file 2 — Fig. S2. N601K mutation in BD4 reduces H3K14ac binding while Y600N601‐AA mutation does not. The indicated Flag‐BD234 constructs were expressed in HEK293T cells, pulled down by the indicated peptides, and immunoblotted with anti‐Flag antibody. [file MOL2-13-811-s002.tif]

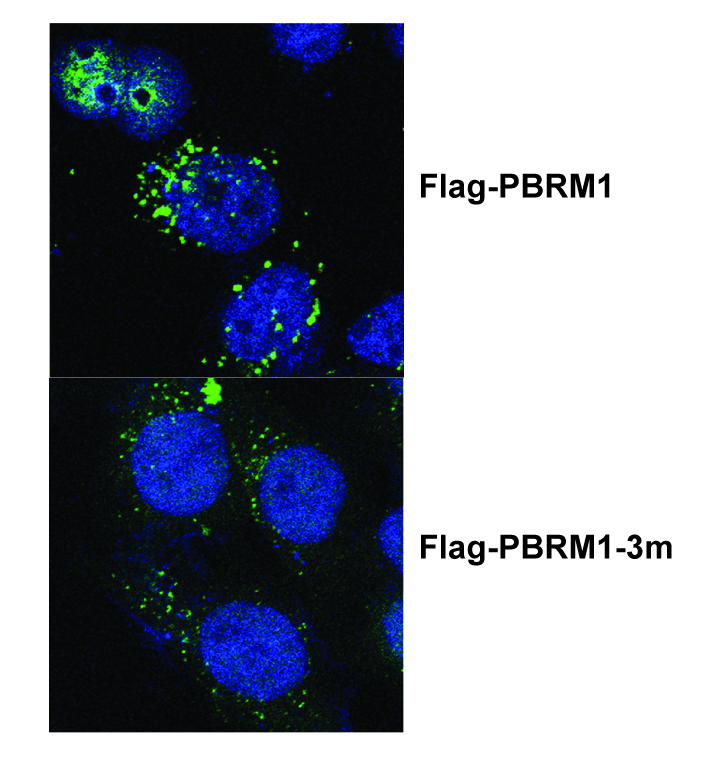

Supplement: Supplementary file 3 — Fig. S3. Concurrent point mutations in BDs 2, 4 and 5 cause PBRM1 to relocalize to the cytoplasm. Flag‐tagged wild‐type or 3m mutant PBRM1 constructs were expressed in HeLa cells. Immunofluorescence was performed using anti‐Flag antibody. The DNA is stained blue with DAPI, while the PBRM1 signal is stained green. [file MOL2-13-811-s003.tif]
